# Reality check: testing the *in silico* predictions of false negative results due to mutations in SARS-CoV-2 PCR assays using templates with mismatches *in vitro*

**DOI:** 10.3389/fcimb.2025.1524025

**Published:** 2025-10-31

**Authors:** Taylor Otwell, Brittany Knight, Michael Coryell, Jennifer Stone, Phillip Davis, Bryan Necciai, Paul Carlson, Shanmuga Sozhamannan

**Affiliations:** ^1^ Integrated Health Surveillance and Diagnostics, MRIGlobal, Kansas City, MO, United States; ^2^ Laboratory of Mucosal Pathogens & Cellular Immunology, Division of Bacterial, Parasitic and Allergenic Products, Office of Vaccines Research and Review, Biologics Evaluation and Research, U.S. Food and Drug Administration, Silver Spring, MD, United States; ^3^ National Strategic Research Institute, Nebraska Medical Center, Omaha, NE, United States; ^4^ Defense Biological Product Assurance Office (DBPAO), Joint Program Executive Office for Chemical, Biological, Radiological and Nuclear Defense (JPEO-CBRND), Enabling Biotechnologies, Frederick, MD, United States; ^5^ Joint Research and Development, LLC, Stafford, VA, United States

**Keywords:** signature erosion, real time PCR, qPCR, SARS-CoV-2, PCR efficiency, *in silico* prediction, false negative (FN), wet lab testing

## Abstract

Molecular diagnostic assays are critical tools to test, diagnose and treat infectious and other diseases. For example, PCR test results have been extremely valuable during the COVID-19 pandemic, not only to provide appropriate health care for infected and symptomatic individuals as needed, but also for implementing public health measures such as test, trace and isolate infected and asymptomatic individuals to prevent further transmission of the virus. Sustained transmission and unhindered proliferation of the pathogen across the population during a continuous, ongoing pandemic such as COVID-19, resulted in many variants with mutations. These mutations may lead to signature erosion, a phenomenon wherein diagnostic tests developed using the genomic sequence of an earlier version of the pathogen, may fail and cause a false negative (FN) result in a sample containing a new variant. We and others have developed applications such as PSET (PCR Signature Erosion Tool) to monitor the performance of diagnostic tests *in silico* using pathogen genomic sequences. Here, we present and discuss the data on wet lab testing of the *in silico* predictions to assess assay performance with mismatches in assay signatures. We found that the majority of the assays performed without drastic reduction in assay performance even with mismatches in primer and probe regions as measured by PCR efficiencies and C_t_ value shifts. We identified critical residues and positions and types of changes that may impact assay performance. Despite the extensive accumulation of mutations in SARS-CoV-2 variants over the course of the various waves of the pandemic, most PCR assays proved to be extremely robust and continued to perform well even with drastic changes and signature erosion.

## Introduction/background

Real time PCR assays are the bedrock of pathogen detection in various clinical, veterinary, and environmental sample types. In these tests, presence of a unique part of a pathogen genome is examined by polymerase chain reaction based nucleic acid amplification. Usually, the target amplicon, a very tiny portion of the genome (e.g., ~0.0018% of *Bacillus anthracis* or ~0.33% of SARS-CoV-2 genome for a 100 bp amplicon), is used as a proxy for the presence of the pathogen. The underlying success of the PCR test relies on specific and efficient binding of the primers and probes to the complementary target nucleic acid sequences present in the sample and subsequent amplification. Several parameters contribute to the efficiency of the PCR which include composition of bases (GC content), the interaction kinetics of the primer and probe to the target sequences reflective of the percent identity between the two components and reaction conditions (ionic strength and other reagents) and cycling parameters. Designing successful PCR assays may be impacted by the many inherent features of the pathogen genome sequences and their diversity and hence availability of contiguous stretches of unique sequences of sufficient length for designing a PCR assay (Stadhouders et al., 2010). In addition, appropriate target selection during assay design is also critical to achieve high specificity of an assay, i.e., the assay should only detect the agent or pathogen of interest and not any near neighbor or any other DNA that may have some identity to the intended amplicon target. For the most part, a well-designed assay detects all strains of a given target pathogen and excludes everything else. The assays are designed based on available sequences of the target agent at a given time and hence may only be effective against the sequence diversity known at that time.

Democratization of next-generation sequencing has enabled the generation of thousands of whole genome sequences of a given pathogen directly from clinical specimens, as evidenced during the SARS-CoV-2 pandemic. As of September 03, 2024, around 16,934,260 sequences have been generated and shared from many countries around the globe via GISAID, the global data science initiative (Khare et al., 2021). The sensitivity, speed, scalability, and reduced costs of modern-day sequencers make whole genome sequencing an attractive diagnostic tool—even rivaling PCR—though there are still a number of challenges that currently prevent whole genome sequencing from completely eclipsing PCR as the predominant diagnostic tool for SARS-CoV-2 and other infectious diseases (John et al., 2021). This genomic revolution has created an opportunity as well as challenges for molecular assay design, development, testing, validation, and continuous performance evaluation and monitoring. With the constant evolution of the given target pathogen over time, there is a possibility that the assay target may show signature erosion and the assays may fail, resulting in false negative results. Hence, real time periodic monitoring of assays *in silico* against newer sequences can potentially reveal such failures in advance. This will enable redesigning of assays to address the changing genomic profile even before a variant becomes dominant and causes overwhelming false negative results in clinical and environmental sample testing. SARS-CoV-2 exemplifies such a scenario and *in silico* monitoring has revealed such failures in diagnostic assays [e.g., S Gene Target Failure (SGTF) from alpha variant] over time during the pandemic (Davies et al., 2021). Mismatch in assay signatures can also be beneficial for discriminating wild-type and variant strains, as evidenced by SGTF. Many studies have reported *in silico* monitoring as a means of assessing assay failures (Sozhamannan et al., 2015; Khan and Cheung, 2020; Miranda and Weber, 2021; Mentes et al., 2022; Rana et al., 2022).

However useful *in silico* monitoring of assays may be, these approaches may not accurately predict assay failures in analytical or clinical sample testing. The assay failure and hence false negative results in wet lab testing may result from not only the type and number of mismatches in signature sequences or position of the mismatches from the priming site but also other parameters such as ionic conditions of the PCR reaction and matrix effects. Many *in silico* predictions do not account for these factors. Understanding the impact of these factors may aid in tweaking the *in silico* approaches to better predict assay failures and may potentially circumvent the need for wet lab testing.

The basic physical parameters of nucleotide mismatches and ionic conditions and their impact on PCR have been well defined (Eun, 1996). In general, a 1% base mismatch reduces the melting temperature (T_m_) by 1.0–1.4 °C (Bonner et al., 1973). For an oligonucleotide, single base pair mismatches can affect the T_m_ by as much as 10 °C (Wallace et al., 1979). When the T_m_ is decreased by ∼15 °C due to mismatches, the annealing rate of the DNA is reduced by a factor of two (Chang et al., 1974). Mismatching hybrids are more stable at high salt than at low salt concentrations, approximately 66% being the minimum match. The stringency of hybridization can be adjusted by several factors such as temperature, ionic strength, and chaotropic agents (Nozari et al., 1986).

There is extensive documentation on wet lab testing the impact of mismatches between primers/probes and templates. Different experimental approaches can be taken to assess PCR assay performance. Priming probabilities, for example, were found to be a good measure of analytical specificity (Boyle et al., 2009; Wright et al., 2014). Another study quantitatively investigated the effects of primer-template mismatches within the 3-end primer region on real-time PCR using the 5-nuclease assay (Stadhouders et al., 2010). The results showed that single mismatches instigate a broad variety of effects, ranging from minor (<1.5 cycle threshold, e.g., A–C, C–A, T–G, G–T) to severe impact (>7.0 cycle threshold, e.g., A–A, G–A, A–G, C–C) on PCR amplification.

A systematic approach to assess the effects of mismatches and their positions from the 3’end was conducted (Lefever et al., 2013). Single mismatches located >5 bp from the 3 end have a moderate effect on qPCR amplification and can be tolerated and complete blocking of the PCR reaction was observed for 4 mismatches. A number of additional studies have drawn similar conclusions (Christopherson et al., 1997; Süss et al., 2009; Wu et al., 2009; Persson et al., 2019; Howson et al., 2020; So et al., 2020; Cao et al., 2022; Wu et al., 2023). These studies in general point to various parameters to be critical in impacting assay efficiencies because of diversities in experimental set up and other factors and outputs measured. A few recent studies have focused on SARS-CoV-2 as examples and the impact of mutations on PCR efficiencies (Vogels et al., 2020; Storey et al., 2021; Chen et al., 2022).

Overall, the forgoing studies are either focused on introduction of different types and numbers of single nucleotide polymorphisms (SNPs) in predetermined tester-chosen locations, or the assays are restricted to one target gene or organism and the data are extrapolated to other assays and scenarios. Thus, the data were derived from a limited set of template mismatches or a single assay target. Also, these studies could not arrive at a single parameter that can predict false negative results. During the COVID-19 pandemic, a large number (276) of molecular diagnostic assays were developed and issued Emergency Use Authorization (EUA) (United States Food and Drug Administration). These assays are generally spread across the entire genome, and there were many variants with mutations falling within these assay signatures.

The availability of a large set of diagnostic assays and millions of SARS-CoV-2 whole genome sequences provided the opportunity to test *in vitro* a number of naturally occurring permutations of different assays and validate the *in silico* predictions. Here we have conducted extensive testing of 16 assays with over 200 synthetic templates spanning the SARS-CoV-2 genome. We assessed the impact of mismatches in primer and probe binding sites on PCR performance by capturing various metrics such as change in melting temperature (ΔT_m_), amplification efficiency, C_t_ values obtained at various template concentrations, and y-intercept. The use of machine learning models trained on these data to predict the impact of template mismatches on PCR assay performance has been reported elsewhere (Knight et al., 2025). Here we present and discuss the companion wet lab testing data for the 16 assays with various mutant permutations of the wild-type template.

## Materials and methods

### Assay selection

Over the course of the COVID-19 pandemic, periodically, we tracked the performance of 43 SARS-CoV-2 PCR assays using an *in silico* analysis tool called PSET (PCR signature erosion tool) against SARS-CoV-2 sequences from the GISAID database^1^. This tool primarily used percent identity between the query sequence (assay signature sequences comprising of primer, probe and amplicon sequence) and the subject sequences from GISAID. If the mismatch percent in either one of the primers or the probe was >10%, then those subject sequences were considered to have potential for causing false negative results in wet lab testing. The 10% mismatch threshold was chosen somewhat arbitrarily and considering some literature data (Lefever et al., 2013) with the logic that primers of 20 nt length can tolerate up to 2 mismatches without significant reduction in assay performance.

**Figure 1:**
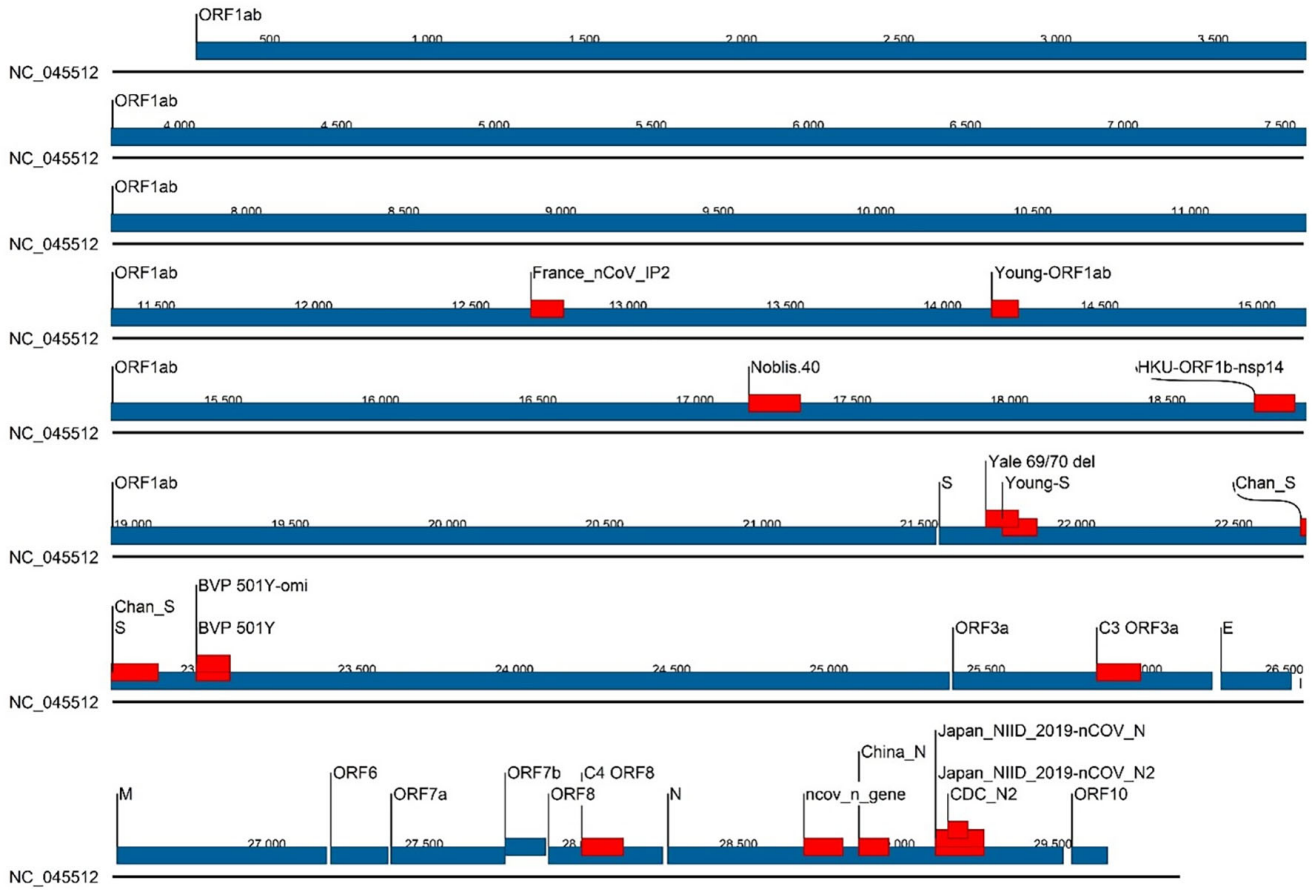
Location of the 16 selected assays on the SARS-CoV-2 genome (NC_045512). Genes are represented by blue bars with annotations, and assay locations by red bars.

Of the 43 assays that were tracked in real time using GISAID sequences, we selected 16 assays for evaluating the accuracy of *in silico* predicted false negative results using synthetic templates with specific mutations in either primer, probe or a combination. These 16 assays are: BVP 501Y, BVP 501Y-omi, C3 ORF3a, C4 ORF8, CDC_N2, Chan-S, China_N, France_nCoV_IP2, HKU-ORF1b-nsp14, Japan_N, Japan_N2, ncov_n_gene, Noblis.40, Yale 69/70 del, Young-ORF1ab, Young-S. Assay designs (primer, probe, and amplicon sequences), sources/references, and genomic locations for all 43 assays are provided in **Supplementary File 1**. Location of the 16 selected assays on the SARS-CoV-2 genome (NC_045512) is displayed in **Figure 1**.

### *In silico* analysis of assay inclusivity and uniqueness of false negative (FN) results

In order to evaluate the inclusivity of each assay, all SARS-CoV-2 genomes designated as “complete” (N = 1793877) were downloaded from NCBI Virus database^2^ (Hatcher et al., 2017) on March 29, 2023, along with a corresponding metadata table containing the Pangolin (Rambaut et al., 2020) lineage assignments. Prediction of amplicon products *in silico*, as well as calculation of inclusivity statistics were performed by the method previously described (Stanhope et al., 2022).

**Figure 2:**
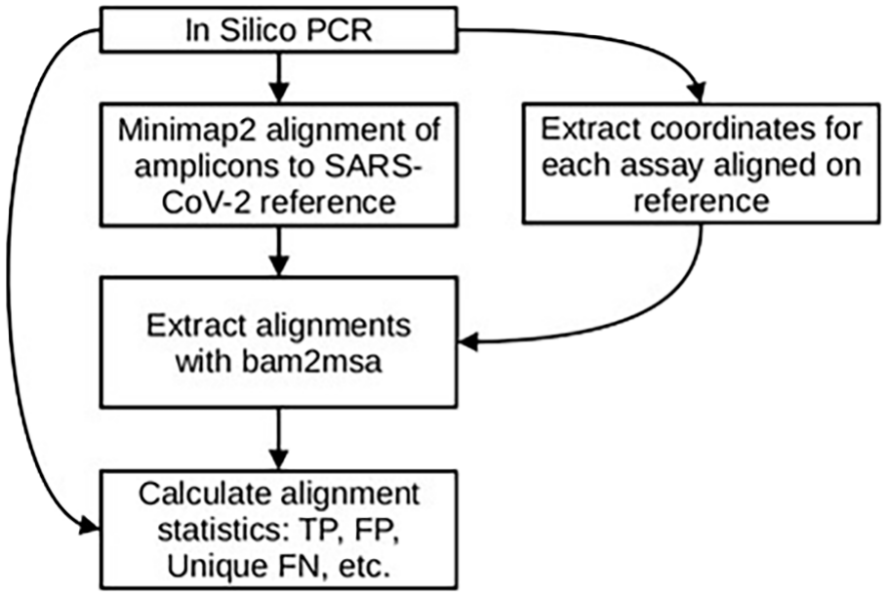
Simulated PCR output is used to generate the expected amplicons used in the alignment step. Coordinates of each assay’s predicted amplicon from the reference genome are used to get intervals for extracting aligned amplicons from the BAM file. These alignments are converted to MSA objects to calculate.

In order to evaluate the number of unique template sequences contributing to False Negatives (FNs) we aligned all of the genomes downloaded for analysis (N = 1793877) to the RefSeq SARS-CoV-2 genome (NC_045512.2) using minimap2 (Li, 2018) with the “asm5” preset corresponding to 5% sequence divergence. For each primer pair we extracted the coordinates from the predicted amplicon table to produce a bed file with the genomic intervals for each assay on the reference genome. Using the BAM file from the minimap2 alignment and the bed intervals for each assay we used bam2msa^3^ to excise all the unique sequence alignments to the region of the reference genome corresponding to each assay. A diagram to illustrate the *in silico* analysis workflow described above is shown in **Figure 2**.

True Positives were calculated from the total number of accession numbers with results in amplicon data table produced by simulate_PCR (Gardner and Slezak, 2014) after the detection filtering criteria of no less than 90% alignment identity across the length of the primers and probes. False Negatives were the set of accession missing after subtracting the True Positive from the total set. While the input number of genomes was 1,793,877, many genomes could not be aligned across various sections of the reference genome for reasons including assembly errors in the query record and large numbers of ambiguous nucleotide characters such as “N”. This resulted in several instances where the number of True Positive detections based on the estimated amplicons from simulate_PCR was larger than the number of assemblies that could be aligned to a region on the reference that corresponds to a primer set. We therefore reported alignment depth in the whole genome alignment at each location. The number of False Negatives in the alignment indicates many false negative sequences were successfully aligned to the reference genome, despite not passing the filtering criteria described above for detection. Unique False Negatives were the count of unique sequences in the Multiple Sequence Alignment (MSA) that came from genomes in the False Negative set for each assay. To calculate the contribution of “N” characters in the input sequences to the number of unique sequences we filtered out any sequences in the MSA that contained an “N” character and then performed the same calculation for Unique False Negatives above for the remaining sequences. For each assay, the number and percentage of aligned sequences identified as False Negatives was calculated for both the full dataset, as well as the dataset after filtering out aligned sequences with “N”s, acknowledging that filtering of these sequences could result in skewing the lineage-level False Negative predictions.

### *In silico* prediction of melting temperatures

For evaluating the determinants of PCR performance, features were engineered to incorporate information about the impact of mismatches on melting temperature of each oligonucleotide, specifically, with candidate template sequences. To produce these features, primer and probe sequences were aligned with the corresponding experimental template sequence. Using the MeltingTemp module from the Biopython library (Cock et al., 2009) melting temperatures of each oligo as it aligns with the experimental templates was calculated with the GC content melting temperature formula: (Tm=81.5+0.41(%GC)−500/N+16.6×log[Na+]/(1.0+0.7×[Na+])) , where Na+ is the millimolar concentration of sodium ions, GC is the fractional GC content of the alignment and N is the length of the oligonucleotide (Wetmur, 1991). The “Annealing Temp Change” features were calculated by subtracting the estimated oligonucleotide melting temperature for each probe and template alignment under consideration from the ideal estimated melting temperature, where the oligonucleotides are exact matches for the template.

### PCR design and experiments

Template sequences selected for wet lab testing are provided alongside a summary of the test results in **Supplementary File 2**. Representative false negative (FN) templates and positive control templates were ordered as synthetic DNA oligos (gBlock fragments) from IDT (Coralville, Iowa), and included 20 bps of flanking template sequence on each end of the amplicon sequence. The FN templates were tested at four levels (50, 500, 5000, and 50,000 copies per reaction) in triplicate reactions alongside no template controls (NTCs) and positive controls (PCs) (wild-type with no mismatches). A universal set of reagents and parameters was used for testing all assays, which included TaqPath 1-Step RT-qPCR Master Mix, CG (Thermo Fisher Cat. No. A15299). Primers and probes (IDT, Coralville, Iowa; PrimeTime™ 5’ 6-FAM™/ZEN™/3’ IB^®^FQ) were included at final concentrations in the reaction of 900 nM and 250 nM, respectively. These concentrations were selected based on the highest concentrations recommended by the manufacturer for this master mix, because higher concentrations enhance the likelihood of primer and probe binding, meaning higher concentrations are often more permissive of mismatches. The final reaction volume was 20 µL, with 5 µL of template added to 15 µL of master mix. The thermal cycling protocol used was as follows: reverse transcription at 50 °C for 15 minutes, initial denaturation at 95 °C for 2 minutes, and 50 cycles of 95 °C for 3 seconds and 55 °C for 30 seconds, with data collection at the end of each cycle. This protocol corresponds to the manufacturer’s recommendations for this master mix, with two modifications: 1) The number of cycles was increased from 40 to 50 to allow generation of C_t_ values for templates with suboptimal amplification efficiency due to mismatches or deletions, and 2) The annealing and extension temperature was reduced from 60 °C to 55 °C to be more permissive of mismatches and reflective of annealing/extension temperature recommended for many of the published assays evaluated.

PCR was performed on a Bio-Rad CFX96 real-time PCR instrument followed by analysis using a universal threshold in order to assess mismatched template performance to the wild-type positive control. In addition to qualitative results (detection or no detection), quantitative performance metrics captured included average C_t_ values at each level tested, amplification efficiency, linear regression coefficient (R^2^), and y-intercept (the theoretical C_t_ value that would be obtained for a single copy of template, which can be used as rough indicator of an assay’s anticipated analytical sensitivity).

## Results

### *In silico* prediction of false negative results

**Figure 3:**
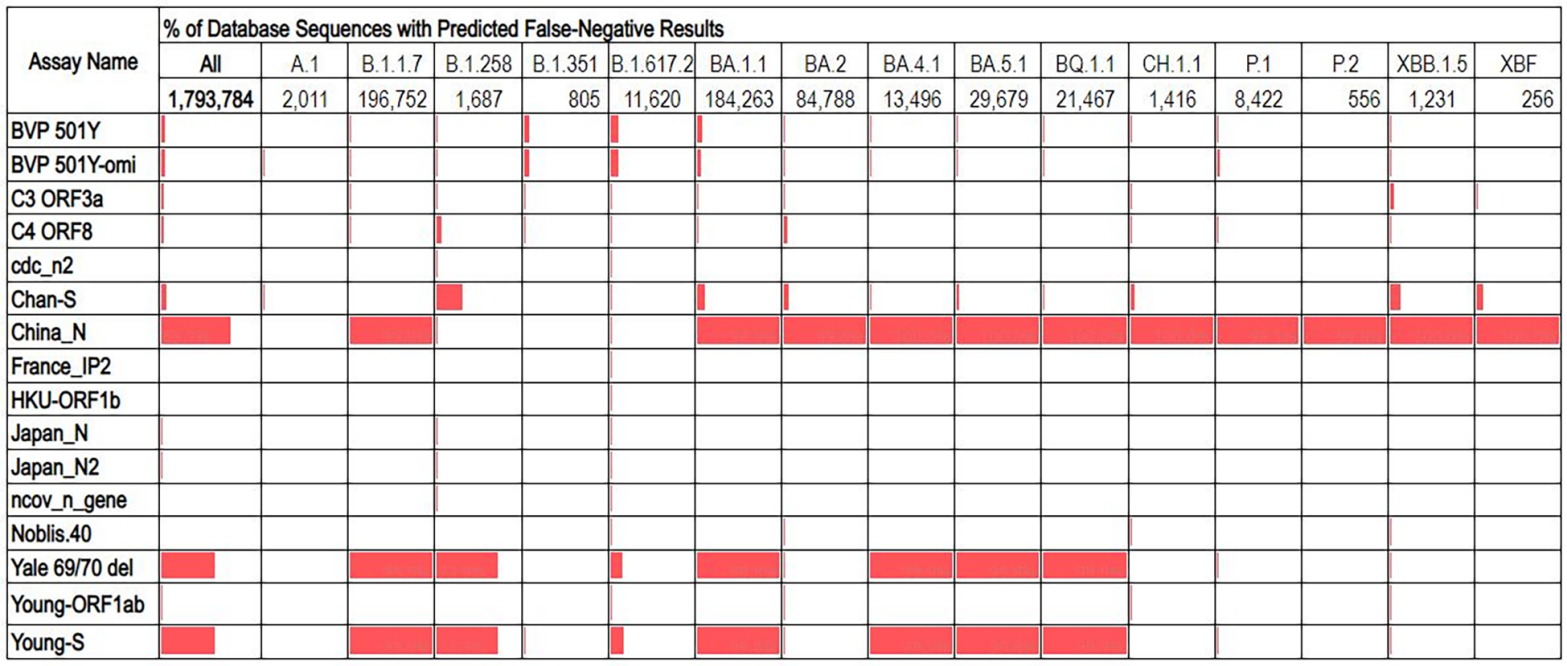
Percentage of database sequences predicted to cause false negative results for each assay, with percentages represented by red data bars. Total number of sequences present in the database for each lineage are displayed below the lineage name.

The overall false negative results predicted for most assays was ≤5%, with three exceptions: China_N, Yale 69/70 del, and Young_S (**Figure 2**). These three assays had overall false negative rates of 69.7%, 54.1%, and 54.2% respectively. Analysis of false negatives for these assays on a per-lineage basis revealed that the overall high false negative results are contributed to primarily by some lineages which produced very high predicted false negatives (99-100%), while other lineages showed false negatives percentages of<1%. Similarly, some assays with low overall predicted false negative results produced moderately high false negatives for some specific lineages. For example, the Chan_S assay had an overall predicted false negative of just 5%, but for some lineages of interest (e.g., B.1.258) were >30% (**Figure 3**).

Because of the heterogeneity of predicted false negative percentages across different lineages, we hypothesized that the majority of predicted false negative results might result from non-unique mutations prevalent in specific lineages. This was investigated by aligning database sequences to the assay regions and determining the percentage of unique false negative sequences aligned (after excluding sequences with ambiguous “N” nucleotides). This analysis revealed that for all assays, only a very small proportion (<2%) of the aligned false negative sequences were unique, regardless of whether the assay had low or high overall percentage of false negative sequences in the alignment (**Table 1**). This may also indicate overrepresentation of specific lineage sequences in the database.

**Table 1:** Results from evaluating the number of unique false negatives for each assay.

Assay name	Alignment depth	Number of false negatives in alignment	Unique False negatives without ambiguous bases (N’s)	% Aligned sequences that are false negatives^1^	% of False negatives that are unique and without ambiguous bases (N’s)^2^
BVP 501Y	1666251	31440	191	1.9%	0.6%
BVP 501Y-omi	1666251	31495	126	1.9%	0.4%
C3 ORF3a	1663699	3193	9	0.2%	0.3%
C4 ORF8	1645532	9842	151	0.6%	1.5%
CDC_N2	1644846	783	8	0.0%	1.0%
Chan-S	1669773	37022	5	2.2%	0.0%
China_N	1644937	1153000	846	70.1%	0.1%
France_nCoV_ IP2	1762228	1964	23	0.1%	1.2%
HKU-ORF1b-nsp14	1746015	1336	15	0.1%	1.1%
Japan_N	1644843	1293	19	0.1%	1.5%
Japan_N2	1644843	1253	5	0.1%	0.4%
ncov_n_gene	1645137	814	13	0.0%	1.6%
Noblis.40	1757050	1121	5	0.1%	0.4%
Yale 69/70 del	1700933	907518	737	53.4%	0.1%
Young-ORF1ab	1760030	4280	0	0.2%	0.0%
Young_S	1700858	907829	713	53.4%	0.1%

^1^Percentage of Aligned sequences that are false negatives = (Number of false negatives in alignment)/(Alignment depth) x 100.

^2^Percentage of False Negatives that are unique and without ambiguous bases = (Number of unique false negatives without ambiguous bases)/(Number of false negatives in alignment) x 100.

### Assay performance with FN templates containing mismatches in primer binding sites

**Figure 4:**
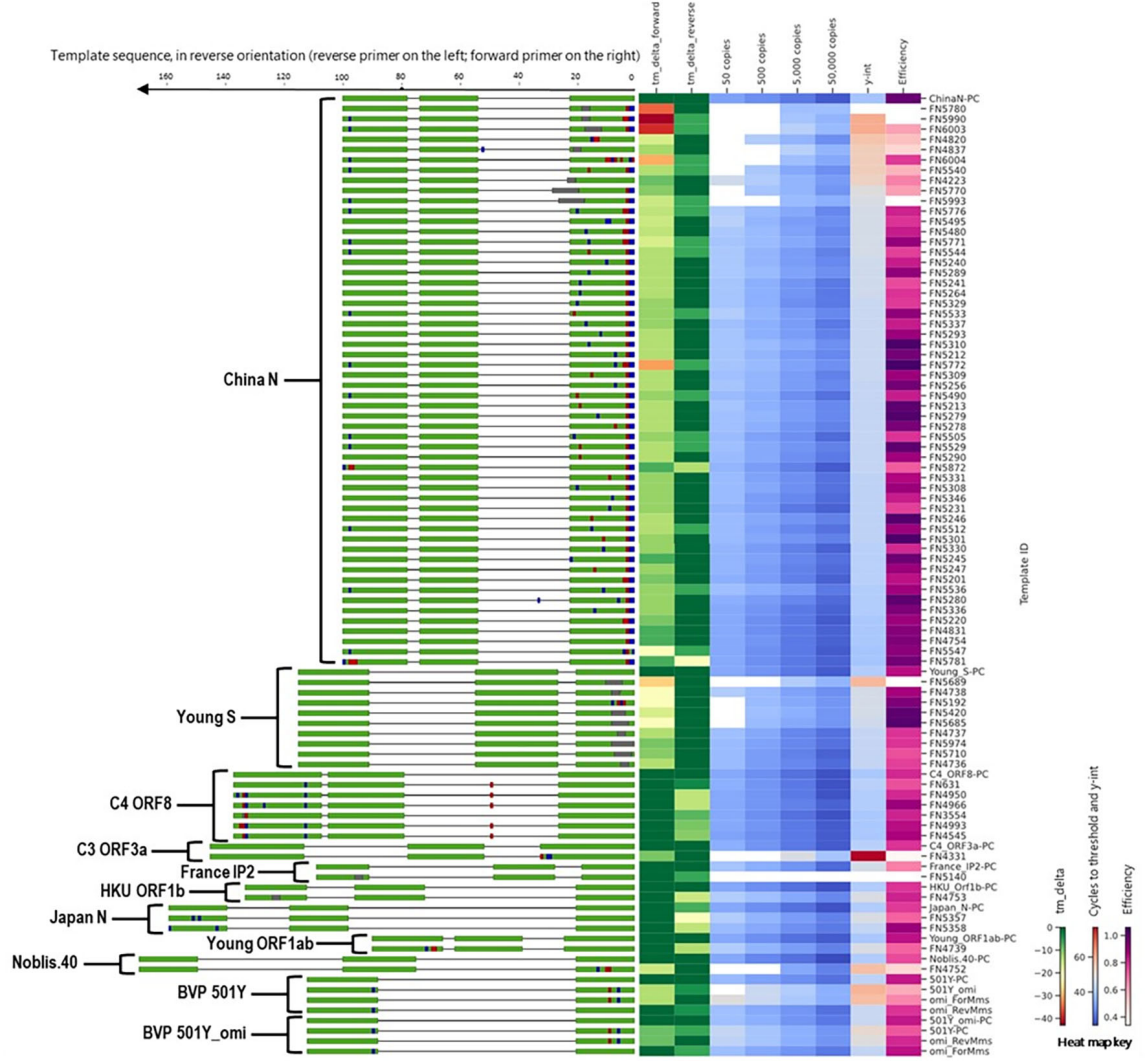
Alignment of all templates with mutations in primer binding sites, alongside a heat map of *in silico* predicted changes in primer T_m_ and *in vitro* PCR testing metrics (average C_t_ values at 50, 500, 5,000, and 50,000 copies per reaction; y-intercept; and amplification efficiency). For template sequences, green = primer and probe binding sites; blue = transition mutations; red = transversion mutations; grey = deletions. For ease of visualization, in the assay map, the forward primer is depicted closer to the heat map on the right side.

To evaluate the impact of mismatches in primer binding sites identified during *in silico* analyses, we assessed a total of 97 templates that included templates predicted to cause false negatives and the corresponding positive controls (no primer or probe mismatches) for 11 different SARS-CoV-2 assays (**Figure 4**). Detailed information and performance metrics tables for all assays are provided in **Supplementary File 2**.

**Figure 5:**
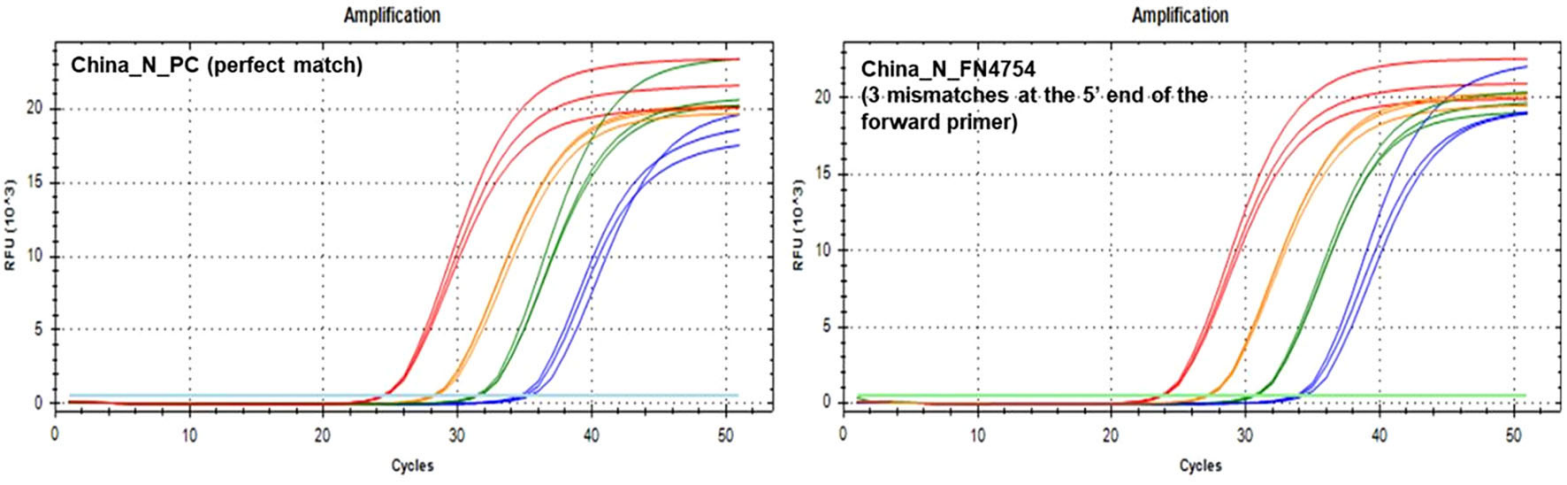
PCR Amplification Plots for the China_N PC template (left) and FN4754 template (right) containing a common 3-nucleotide mismatch in the forward primer region.

During *in silico* analyses, the China_N assay generated the highest overall false negative percentages. This is predominantly due to 3-nucleotide mismatches present at the very beginning (5’ end) of the forward primer (GGG → AAC) common in some lineages (e.g., P.1, P.2, B.1.1.7, and various omicron lineages) that resulted in a<90% match. However, PCR testing with a template containing these 3-nucleotide mismatches (FN4754) produced amplification curves (**Figure 5**) comparable to those produced by the positive control (PC) template containing no mismatches. The absence of a negative impact (contrary to *in silico* predictions based on % match), is likely due to; 1) The 3-nucleotide mismatch is located at the 5’ end of the primer and had negligible impact on amplification, and 2) Despite a decrease in predicted T_m_ of ~7 °C, the predicted T_m_ with the mismatches (56 °C) is still above the annealing and extension temperature (55 °C) used in this study.

Along with the FN4754 template containing the common 3-nucleotide mismatches, 54 additional unique FN templates of the China_N assay were also tested. Summary PCR metrics (amplification efficiency, y-intercept, and average C_t_ values obtained at each test level) for each of these templates are shown as heat maps (**Figure 4**), along with the decrease in *in silico* predicted T_m_ caused by mismatches in the primer binding sites. Only 9 of the 55 predicted China_N FN templates produced false negative results at the lowest template level tested (50 copies per reaction), though many produced delayed amplification, as evidenced by later C_t_ values and y-intercepts.

Another one of the three assays that generated high overall false negative percentages during *in silico* analyses was the Young-S assay. This was primarily due to a high number of sequences containing a 6-nucleotide deletion that is prevalent in many lineages. We tested a total of nine Young-S FN templates, including an FN template (FN5685) with the prevalent 6-nucleotide deletion. Summary PCR metrics (amplification efficiency, y-intercept, and average C_t_ values obtained at each test level) for each of these templates are shown (**Figure 4**), along with the decrease in *in silico* predicted T_m_ caused by mismatches in the primer binding sites. Four of the nine FN templates tested—including FN5685—produced false negative results at the lowest level tested (50 copies per reaction), and all produced delayed amplification (right C_t_ shift) relative to the PC. Interestingly, some templates with similarly sized and even larger deletions than FN5685 produced better PCR results. For example, FN5974 contained a larger 9-nucleotide deletion overlapping the predominant 6-nucleotide deletion, but produced earlier amplification than FN5685 and produced no false negative results at the lowest level. This is because the 9-nucleotide deletion in FN5974 in fact results in just three mismatches in the primer-binding region due to similarities in the upstream flanking region, making the 9-nucleotide deletion less impactful in PCR performance than 3-and 6-nucleotide deletions in the same region. This is also evident in the predicted Δ T_m_ values, with the larger FN5974 deletion causing a Δ T_m_ of 10 °C, while the smaller FN5685 deletion caused a Δ T_m_ of 21 °C.

We tested six C4 ORF8 FN templates, each containing up to four mismatches in the reverse primer (**Figure 4**). None of the tested FN templates produced notably delayed amplification, with maximum C_t_ shifts of around 1–2 C_t_ values. We also tested one C3 ORF3a FN template, which contained three mismatches in the forward primer. In contrast to the minimal impact observed for C4 ORF8 templates with up to four mismatches in a primer binding site, the C3 ORF3a FN template with three mismatches caused significant amplification delays, resulting in failed detection below 5,000 copies per reaction and large right C_t_ shifts (>15 C_t_ values) at higher test levels. The extreme negative impact is likely attributed to the position of the three mismatches, which are all located within the last four nucleotides of 3’ end of the forward primer.

Additionally, we tested one France_nCoV_IP2 FN template, which contained a 3-nucleotide deletion near the 3’ end of the reverse primer and caused false negative results at all levels tested. We also tested one HKU-Orf1b-nsp14 FN template that contained a 3-nucleotide deletion near the middle of the reverse primer and two Japan_N FN templates with two mismatches each in the reverse primer region, one Young-Orf1ab FN template with three mismatches in the reverse primer region, and one Noblis.40 FN template with three mismatches in the forward primer region. All of these FN templates produced significantly delayed amplification (C_t_ shifts of 3–8 C_t_ values), but only the Noblis.40 FN template produced false negative results, and only at the 50 and 500 copies/reaction levels.

### Assay performance with FN templates containing mismatches in probe binding sites

**Figure 6:**
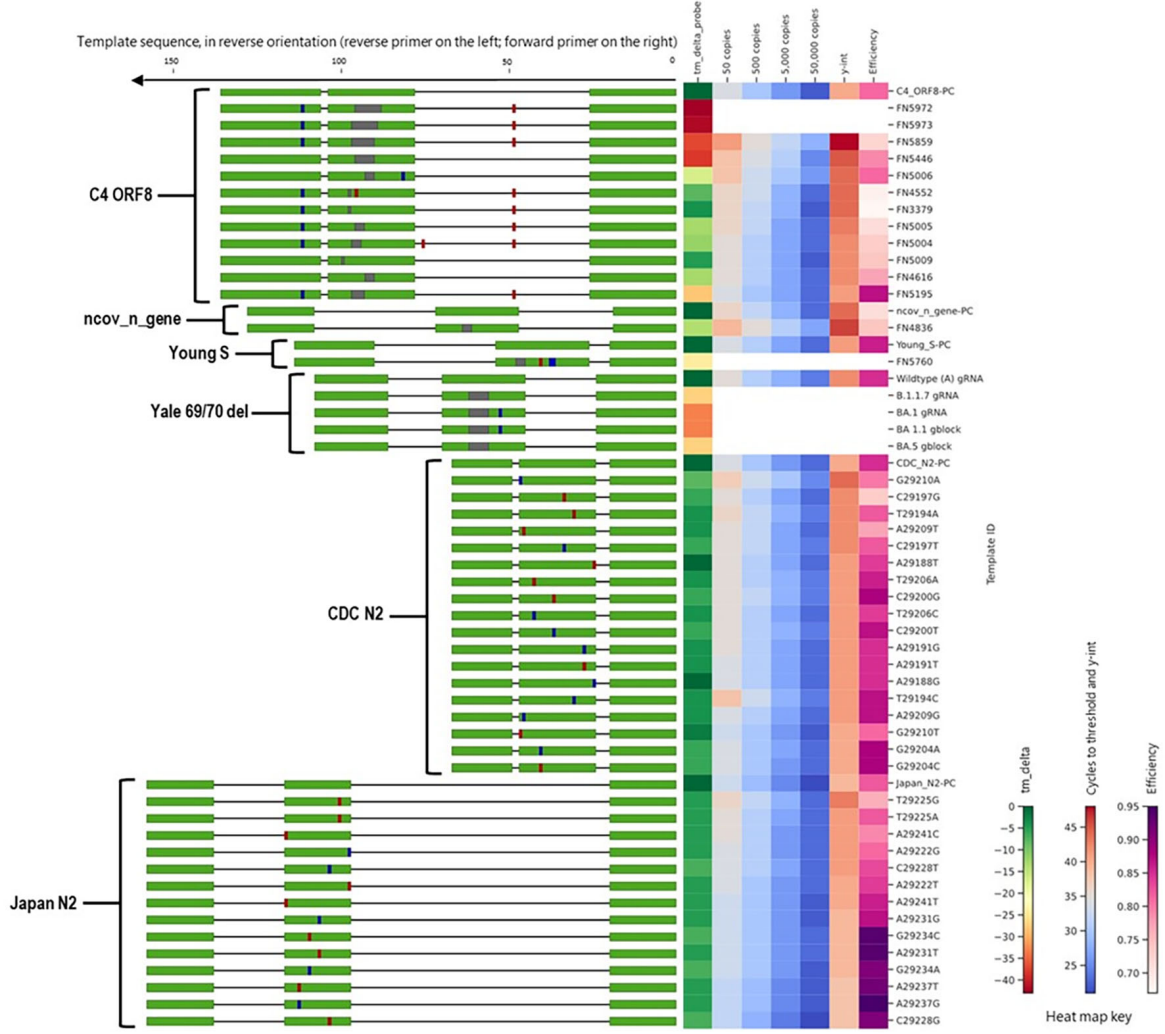
Alignment of all templates with mutations in probe binding sites, alongside a heat map of *in silico* predicted changes in primer T_m_ and *in vitro* PCR testing metrics (average C_t_ values at 50, 500, 5,000, and 50,000 copies per reaction; y-intercept; and amplification efficiency). For template sequences, green = primer and probe binding sites; blue = transition mutations; red = transversion mutations; grey = deletions. For ease of visualization, in the assay map, the forward primer is depicted closer to the heat map on the right side.

To evaluate the impact of mismatches in probe binding region, we tested a total of 56 templates, that comprised of templates predicted to cause false negatives during *in silico* analyses and the corresponding positive controls (no primer or probe mismatches) for 6 different SARS-CoV-2 assays (**Figure 6**).

We tested ten FN templates derived from one assay, C4 ORF8, with deletions in the probe region (**Figure 6**). This assay demonstrated a surprisingly high tolerance to deletions in the probe binding region. Assay performance was only moderately affected for templates with a deletion of ≤6 nucleotides (of a 26-nucleotide probe binding site), with C_t_ shifts of ≤5 C_t_s and no failed detection (false negatives), even at the lowest template concentration (50 copies/reaction). For the FN template with a 7-nucleotide deletion (FN5859), the C_t_ shift was more pronounced and the average C_t_ value at 50 copies/reaction was >40. For the two FN templates with 8-nucleotide deletions (FN6972 and FN5973), no positive results were produced at any of the template levels tested.

Additionally, we tested one ncov_n_gene FN template, which contained a 3-nucleotide deletion in the probe region (**Figure 6**). This template did not produce false negative results at any levels tested, although it did cause C_t_ shifts of approximately 3 C_t_ values at each level tested. We also tested one Young-S FN template that contained a 3-nucleotide deletion in the probe region and three mismatches; this template produced false negative results at all levels tested.

### Comparison of the impact of mismatches between primer and probe binding regions

**Figure 7:**
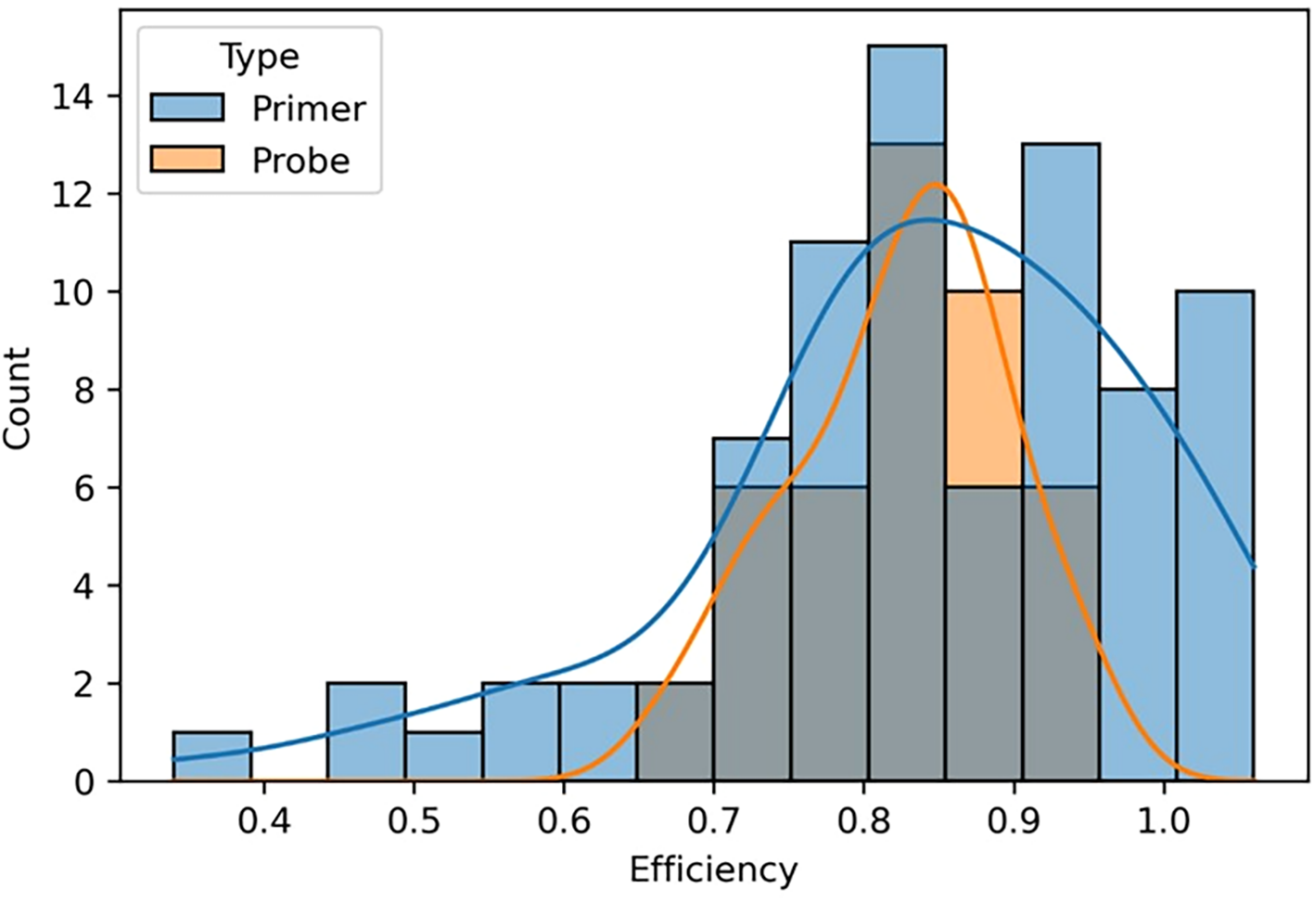
A graphical representation comparing the effects of mismatches in primer and probe binding regions on amplification efficiency. The gray portion of the bar graph represents assays containing both primer and probe mismatches. Counts represent the number of templates that fell within each efficiency value grouping.

We compared the overall impact of mismatches in primer versus probe binding regions on amplification efficiency and found that mismatches in primer binding regions produced a much broader spectrum of amplification efficiencies, whereas mismatches in probe binding regions had a narrower effect (**Figure 7**). Probe mismatches were more likely to result in a weaker fluorescent signal rather than reduced amplification efficiency, producing a more binary qualitative result of either detection or no detection depending on the impact. These data collectively suggest that primer and probe binding region mismatches should be considered individually when evaluating the potential for assay failure and false negative results.

### Variant panel assay that produced unexpected false negative results not predicted by the *in silico* pipeline

**Figure 8:**
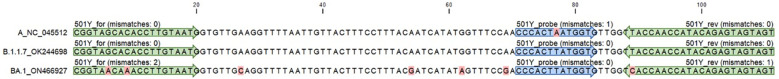
Alignment of the BVP 501Y amplicon region for A (wild type), B.1.1.7, and BA.1 reference sequences. Green = primer binding site; blue = probe binding site; red = mutation or mismatch.

The N501Y mutation is present in many lineages, and we previously developed a triplex panel that included this mutation for detecting variants (Stanhope et al., 2022). This assay is specific for the mutant allele and PCR amplification is seen only with the mutant template and not with the wild-type template. *In silico* analyses of the presence of N501Y in omicron and its sub lineages indeed showed the presence of the mutation in these lineages (BA1, BA1.1, BA2, BA3, BA4, and BA5). However, this assay produced false negative results in retrospective testing with Omicron clinical specimens. Alignment of the amplicon region for reference sequences from wild-type (A), B.1.1.7, and Omicron BA.1 strains showed that the Omicron variant had mutations in both primer regions: two mutations in the forward primer and one in the reverse primer (**Figure 8**). However, the *in silico* inclusivity analysis pipeline did not identify this as a potential false negative, as two mismatches in a 20-nucleotide primer is below the ≤10% mismatch threshold that triggers classification as a false negative.

To investigate whether the false negative result was due to mismatches in the forward or reverse primer binding site, or a combination of both, three synthetic templates were tested: one with all three mutations present in Omicron primer regions (“omi_FN”), one with only the forward primer mismatches (“omi_ForMms”), and one with only the reverse primer mismatch (“omi_RevMms”), as previously shown in **Figure 3**. The resulting PCR metrics indicated that the two mismatches in the forward primer are responsible for the delayed amplification and resulting false negative results (see **Table 2**). The reverse primer mismatch has little effect, despite being located near the 3’ end.

**Table 2:** Average C_t_ values obtained with the original 501Y assay and the 501Y-Omicron assay, demonstrating significant adverse impacts of two forward primer mismatches and minimal impacts of the one reverse primer mismatch.

Assay	Template	No. of mismatches		Avg. C_t_ value (n=3)			
		Forward primer	Reverse primer	50 copies	500 copies	5,000 copies	50,000 copies
BVP 501Y	B.1.1.7 PC	0	0	37	33	30	26
BVP 501Y	Omicron PC	2	1	ND	45	41	35
BVP 501Y	omi_RevMms	0	1	38	34	31	26
BVP 501Y	omi_ForMms	2	0	49	45	40	35
BVP 501Y-omi	B.1.1.7 PC	2	1	44	39	35	31
BVP 501Y-omi	Omicron PC	0	0	38	35	31	27
BVP 501Y-omi	omi_RevMms	2	0	43	39	35	31
BVP 501Y-omi	omi_ForMms	0	1	38	34	31	27

### Testing of Alternative 501Y Primers

We designed new primers to specifically detect the N501Y mutation in Omicron strains. When aligned to the B.1.1.7 sequence, the new assay (“501Y-omi”) contained two mismatches in the forward primer region and one mismatch in the reverse primer region (**Figure 4**). This assay was tested with the same set of four templates previously tested with the original 501Y assay (**Table 2**). The new assay produced good results with the perfectly matched Omicron template and the template containing just one mismatch in the reverse primer but produced a notable C_t_ shift (approximately 4–6 C_t_ values) when used with the B.1.1.7-derived PC for the original assay and the other template containing two mismatches in the forward primer region. Similar to results obtained for the original BVP 501Y assay, results for the 501Y-omi assay confirmed that having just two mismatches in the forward primer region was significantly detrimental to assay sensitivity, despite being below the ≤10% mismatch threshold used to identify potential false negatives *in silico*, and that the mismatch in the reverse primer had negligible impact, despite being just one nucleotide from the 3’ end.

**Figure 9:**
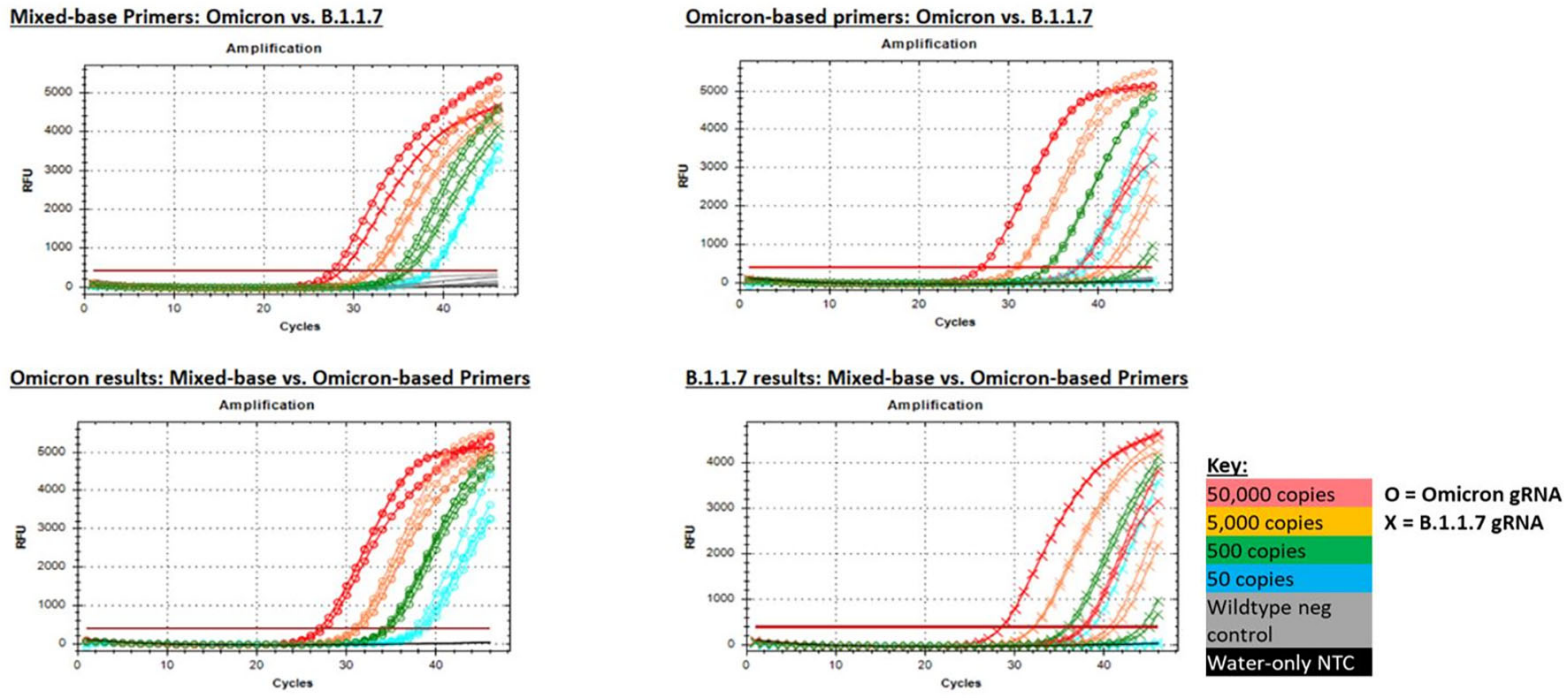
PCR amplification plots for the 501Y mixed base primers and Omicron-based primers (501Y-omi) when tested with gRNA from Omicron and B.1.1.7 strains. Mixed base primers produced comparable results for both variant strains (top left), whereas Omicron-based primers produced strong detection of Omicron gRNA, but significantly delayed amplification of B.1.1.7 gRNA that resulted in failed detection at the lowest test level (top right). Likewise, both primer sets produced comparable results for Omicron gRNA (bottom left), whereas only the mixed base primers produced strong detection of B.1.1.1 gRNA (bottom right).

As an additional option for detection of the N501Y mutation, we designed primers containing mixed bases at the three nucleotide locations in the original primer set that were mismatched with the Omicron sequence. Mixed-base primers allowed comparable detection of both Omicron and the original variants (represented by B.1.1.7), whereas the Omicron-based primers (501Y-omi) allowed strong detection of Omicron but delayed (though not eliminated) detection of B.1.1.7 (**Figure 9**). Based on these results, we concluded that mixed-base primers could be used for broader detection of the 501Y mutation in both Omicron and non-Omicron variants.

### Assays with known failures in clinical testing

We tested three assays known to produce negative results with some variants in clinical testing: the Yale 69/70 del assay (which is known to produces negative results for variants containing the S:60/90 deletion), the CDC_N2 assay, and the Japan_N2 assay.

### Yale 69/70 del (SGTF) assay

**Figure 10:**
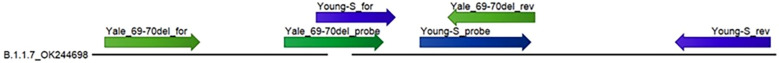
Alignment of the S:Δ69/70 genomic region showing location of the Yale 69/70 del assay sequences (green) and Young-S assay sequences (blue) relative to the 6-nucleotide deletion (represented by a break in the reference line).

In December 2020, the emergence and rapid spread of the Alpha variant was witnessed by the entire global community. Among the many mutations in Alpha variant, a deletion of 6 base pairs in the spike gene resulting in the deletion of two amino acids at positions 69–70 was responsible for some commercial testing kits—e.g., the Thermo Fisher TaqPath COVID-19 assay—producing false negative results in samples containing SARS-CoV-2 Alpha variant. Extensive genome sequencing data confirmed that the widespread S gene target failure (SGTF) phenomenon was primarily due to the new Alpha variant (Public Health England, 2020). The primer and probe sequences of the commercial TaqPath assay are not published; however, the Grubaugh lab published an assay that recapitulates the assay, designated here as the Yale 69/70 del assay (Vogels et al., 2021). Of note, the 6-nucleotide deletion that produces negative results with the Yale 69/70 del assay is the same deletion responsible for the many false negative results predicted for the Young-S assay, but the location of the deletion relative to the primer and probe binding sites is different. With the Yale 69/70 del assay, the deletion is located in the probe region, whereas with the Young-S assay, the deletion is located in the forward primer region (**Figure 10**). As previously discussed, this deletion caused delayed amplification for the Young-S assay and produced false negative results at the lowest level tested (50 copies per reaction).

We tested the Yale 69/70 del assay with five templates representing sequences from five different variant lineages: A (wild type), B.1.1.7, BA.1, BA1.1, and BA.5. All lineages with the common 6-nucleotide deletion in the probe binding site produced negative results at all levels tested, as previously shown (**Figure 6**).

**Figure 11:**
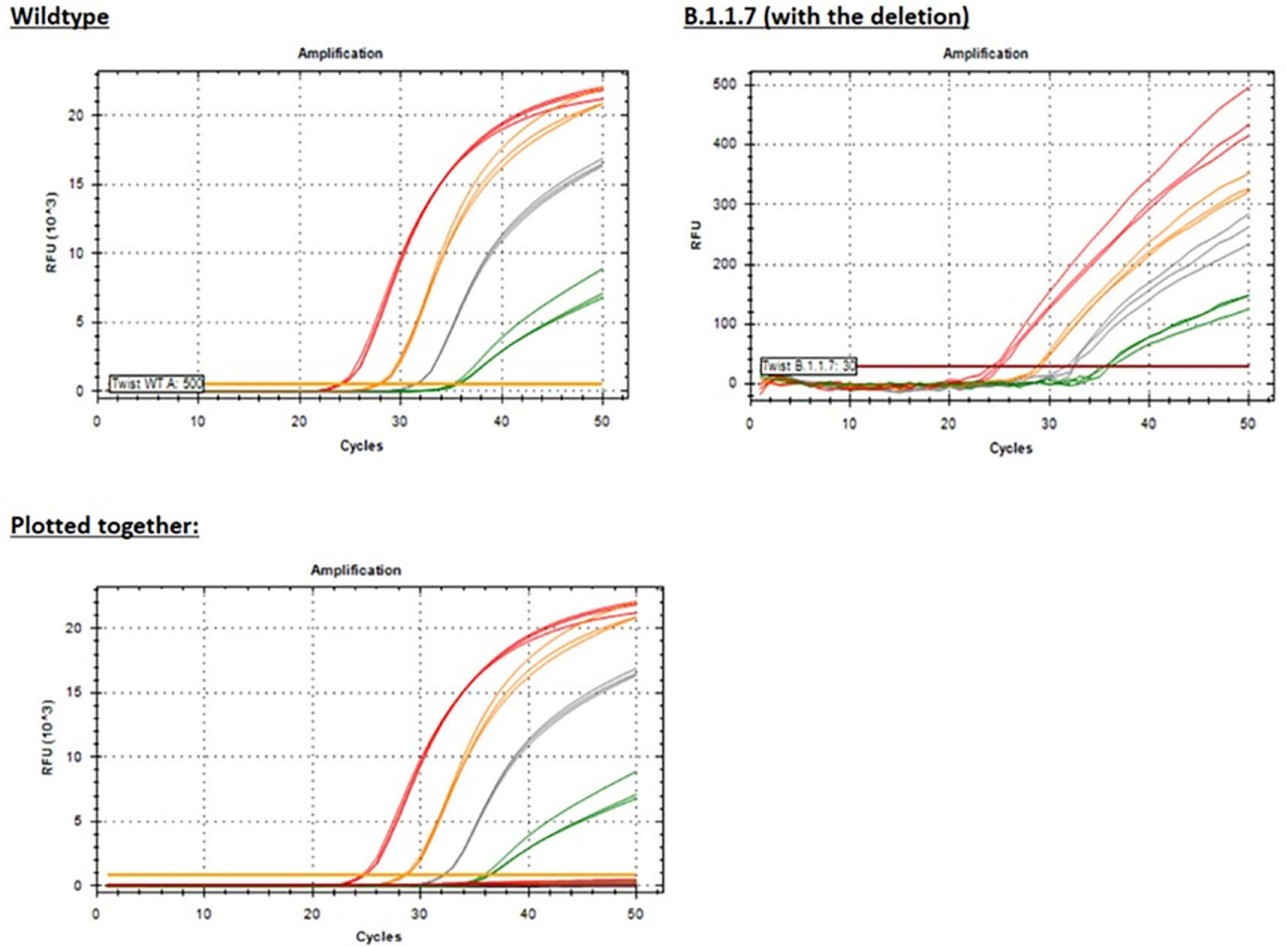
PCR amplification plots for the Yale 69/70 del assay showing results for just the Wild-type template or the B.1.1.7 (with the deletion) template or plotted together for both templates for additional context given the significantly reduced RFU (see the y-axis scale) for the B.1.1.7 template. Red = 50,000 copies/reaction; orange = 5,000 copies/reaction; grey = 500 copies/reaction; green = 50 copies/reaction.

Using white, fluorescence-focusing PCR plates, we were able to detect dim (low RFU) amplification curves for the B.1.1.7 template (**Figure 11**) with Ct values similar to the wild-type template (**Figure 11**), but this would most likely result in false negative results in routine testing of clinical specimens. When plotted together with the positive wild-type template to provide additional context, the B.1.1.7 template amplification is indiscernible from the baseline (**Figure 11**) due to inefficient binding of the probe.

### CDC_N2 and Japan_N2 assays

We tested two additional assays that were producing false negative results in clinical sample testing (Fox-Lewis et al., 2021; Rajib et al., 2022): the CDC_N2 assay and the Japan_N2 assay with mutated probe binding sites. Mutant template tested and the corresponding qPCR results are shown above (**Figure 6**). In contrast to results reported with clinical samples, none of these templates produced false negative results even at the lowest level tested (50 copies/reaction) or exhibited a severe decrease in assay performance (C_t_ difference > 3) using our test method. Differences in master mix reagents, primer and probe concentrations, cycling protocols, instrumentation, and other factors may account for the difference in reported impact of these mutations in clinical specimens versus the lack of impact observed in our analytical testing using synthetic templates.

### RNA templates

**Figure 12:**
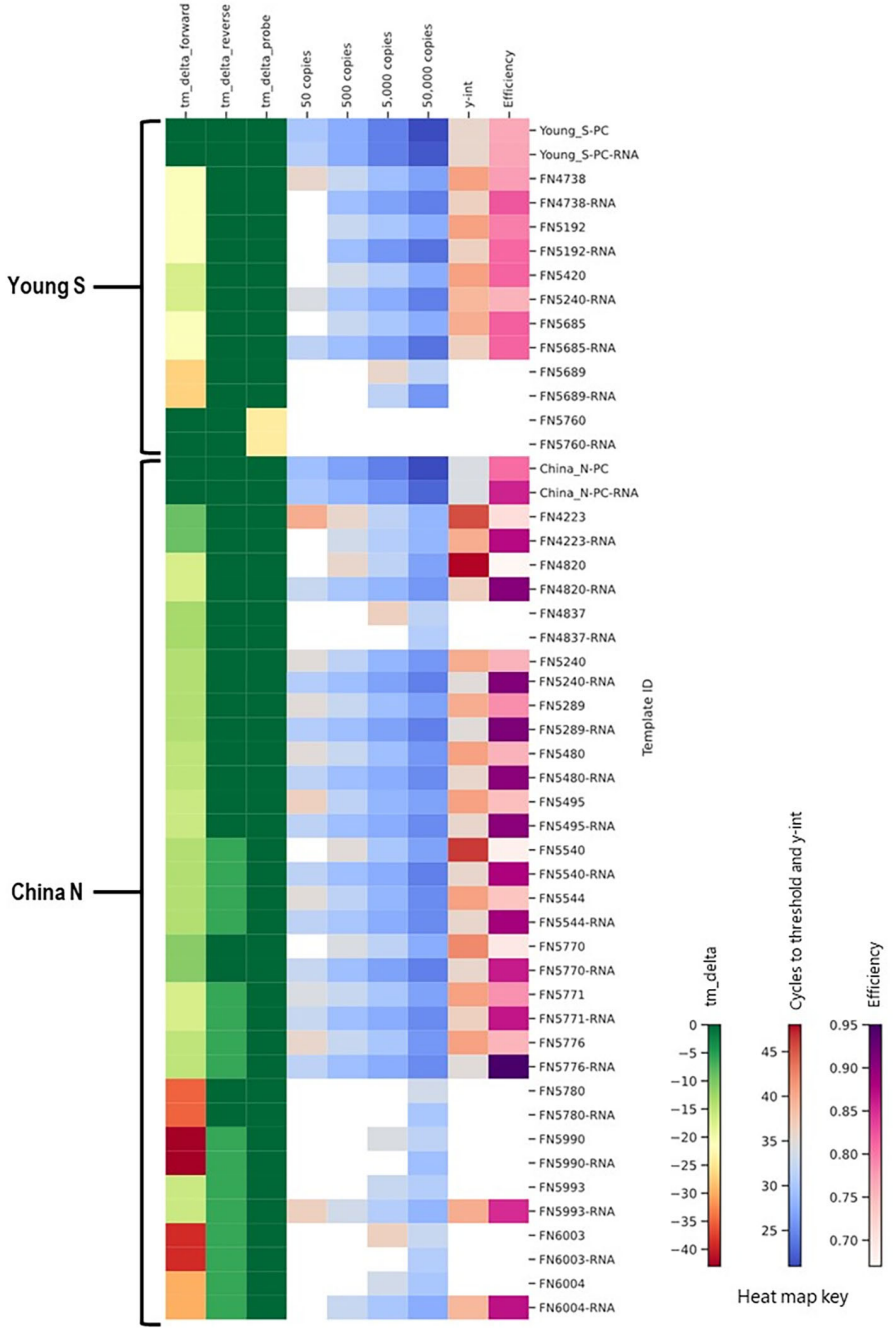
Comparison of results for DNA and RNA templates in a heat map showing *in silico* predicted changes in primer and probe T_m_ and *in vitro* PCR testing metrics (average C_t_ values at 50, 500, 5,000, and 50,000 copies per reaction; y-intercept; and amplification efficiency).

The majority of the foregoing work was performed using synthetic double stranded DNA templates. In order to assess the true impact of mismatches in an assay format where a reverse transcription step is included, we also synthesized and tested RNA templates. A subset of China_N and Young_S templates that demonstrated significantly delayed amplification (y-intercept > 46 or not determined due to amplification failures) during initial testing with DNA templates were synthesized and tested as RNA templates (RNA Ultramers from IDT, Coralville, Iowa) alongside corresponding PC RNA templates. In cases where the primary issue identified by *in silico* analysis was related to a primer mismatch or deletion (as opposed to an issue with the probe), RNA templates typically produced better results than the corresponding DNA templates, as evidenced by lower C_t_ values, lower y-intercepts, and/or lower limit of detection (**Figure 12**). For the one template (FN5760) with a probe issue rather than a primer issue, no difference in performance was observed between the DNA and RNA templates; both template types produced false negative results at all levels tested.

These results underscore the importance of considering template type (DNA or RNA) in assessing the potential impact of mismatches, especially in primer binding regions. The finding that mismatches in primer binding regions have less of an adverse impact on assay efficiency when the template is RNA is likely because RNA templates are initially reverse transcribed prior to amplification during qPCR. Reverse transcription is performed at a lower temperature that is more permissive of binding to templates with mismatches. For single-stranded RNA templates, whether the mismatch is located on the forward or reverse primer may also affect the impact of mismatches, as only one primer is involved in reverse transcription. Furthermore, if reverse transcription successfully occurs from the region with adverse mismatches, the mismatches will be “corrected” by incorporation of the primer sequence in the cDNA produced by reverse transcription.

## Discussion

*In silico* analysis is a widely used and a critical tool to evaluate inclusivity and exclusivity of PCR assays. It is an especially valuable tool for PCR assays designed to detect or diagnose widespread and rapidly evolving pathogens of global concern, such as the SARS-CoV-2 virus. In an earlier study, we reported *in silico* prediction of assay performance and impact of mutations (signature erosion) in *Ebolavirus* molecular assays (Sozhamannan et al., 2015). A number of recent studies have performed *in silico* analyses of SARS-CoV-2 diagnostic assays using genome sequences (Khan and Cheung, 2020; Mentes et al., 2022; Negrón et al., 2022; Rana et al., 2022). These studies have taken only the alignment-based mismatches between primers and probe sequences and not any other PCR parameters into consideration for determining the impact. The accepted rule is that mismatches are detrimental because they alter the primer/probe-template hybridization temperatures and interaction kinetics. An *in silico* approach based on thermodynamic evaluation of the impact of DNA mismatches in PCR-type SARS-CoV-2 primers and probes has been done in one study (Miranda and Weber, 2021). These authors performed a more quantitative assessment of the mismatches on assay performance by considering mismatched hybridization temperature within a range of 5 °C to the fully matched reference temperature. Based on their analyses they recommended to consider mismatch hybridization for the design of primers whenever possible, especially to avoid undesired cross-reactivity. Our study also indicates that Δ T_m_ is a reliable factor that impacts assay performance among various metrics of real time PCR (e.g., efficiency, y-intercept, and C_t_ shift).

Routine assessment of performance *in vitro* or in clinical samples is not feasible in an ongoing outbreak due to delays in real time sample acquisition. In these scenarios, synthetic templates may help, but an improved algorithm may be even more helpful to quickly evaluate assay failures in a changing pathogen genomic profile. In order to achieve that goal, empirical data based on an array of assays, mismatches and varying positions with respect to priming site (3’) and different mismatch types (SNPs and indels) are needed to train a model and incorporate useful features. In this study, we conducted an extensive study on a variety of templates to gather such data using a universal set of test conditions (same master mix, primer and probe concentrations, cycling protocol, and instrumentation) rather than the unique optimized conditions developed and validated for each of the individual assays. It is important to note that we chose this study design with the sole intent of gathering broadly applicable feedback on *in silico* test parameters in a manner that excludes (to the greatest extent possible) the variables inherent in differing or optimized wet lab test conditions. This study was not intended to evaluate clinical test performance of the individual assays evaluated and does not account for variables like assay-specific test conditions, clinical matrix effects, the differences between genomic and synthetic templates, and the use of multiplexed assays to enhance robustness against genetic drift and mutations. While we believe this study design approach is ideal for assay-agnostic *in silico* tool development, we also recognize the limitations of this approach regarding extrapolation to clinical assay performance, and we believe assay-specific test conditions should be incorporated whenever possible into both *in silico* monitoring and wet lab performance testing of established assays.

Over 16 million SARS-CoV-2 sequences have been generated spanning the entire gamut of variants that emerged during the pandemic. We looked at the unique variation permutations across the assay signature sequences and found that unique FNs were extremely low (<0.1 -1.5%) across the 16 assays examined in this study. Among these FNs, we evaluated the laboratory performance of over 200 templates predicted *in silico* (or reported in the literature) to cause false negative results. We found that in some cases, *in silico* analysis accurately predicted failures, but in many instances, templates that were predicted to produce false negative results instead produced PCR results comparable to templates without any mutations, or caused only minor right shifts in C_t_ values, indicating that the assays are more robust to genetic drift than anticipated (or at least *can be* more robust than predicted, depending on the specific wet lab conditions used). Conversely, we also identified cases in which negative, or significantly delayed amplification was observed for templates not identified by *in silico* analysis as potentially problematic templates. In addition, assays that failed in clinical sample testing performed well without any reduction in efficiency in analytical testing with synthetic templates.

This work underscores the importance of performing laboratory testing to confirm or refute *in silico* predictions. It also highlights the need for a better understanding of the factors contributing to whether a given set of mutations will or will not significantly impact assay performance, which in turn could lead to the development of more accurate *in silico* analysis pipelines. To that end, the results obtained in this study suggest that some of the potentially important features to consider include, but are not limited to: 1) Whether the mutations are located in primer or probe binding sites, as that directly impacts the mechanism of potential failure (failed or delayed amplification caused by primer mismatches, versus weak fluorescent signal caused by inefficient probe hybridization), 2) Location of the mutations (i.e., distance from the 5’ vs 3’ end of primers), 3) Type of template (DNA or RNA), 4) Impact of the mutations on melting temperature and hybridization kinetics, and 5) Specifics of the test method being used (e.g., master mix components, primer and probe concentrations, thermal cycling protocol, and PCR instruments used). Based on the data, we have generated a random forest model to assess the impact of mutations on assay performance (Knight et al., 2025). In future work, we will test the model predictions with new designs of an existing poorly performing assay to assess the performance improvement.

Understanding the impact of mismatches on PCR performance may aid in tweaking the *in silico* approaches of building a robust model to better predict assay failures, and may circumvent the need for expansive and expensive wet lab testing. Increased understanding of the impact of mutations in primer and probe binding regions as well as in other parts of the amplicon can not only improve the prediction models used to assess mutation impact during assay design, but also during real world testing of a designed assay in clinical samples containing a variant of the pathogen with specific mutations in the assay signatures. Our future studies will focus on incorporation of data from testing templates with systematically selected or introduced mutations to capture a more comprehensive dataset for inclusion in our *in silico* prediction model, and on more direct validation of our model predictions in analytical and clinical testing scenarios of new assay designs based on the model.

## Data Availability

The raw data supporting the conclusions of this article will be made available by the authors, without undue reservation.

## Footnotes

1
https://gisaid.org/.

2
https://www.ncbi.nlm.nih.gov/labs/virus/vssi/#/.

3
https://github.com/orangeSi/bam2msa.

## Author disclaimers

The content of this publication are the authors’ opinions and do not necessarily reflect the views or policies of the U.S. Department of Defense, the U.S. Department of the Army, or the institutions and companies affiliated with the authors, nor does the mention of trade names, commercial products, or organizations imply endorsement by the U.S. Government. The author (s) reports no potential conflicts of interest. Some authors of this work are military service members or federal/contracted employees of the United States government. This work was prepared as part of their official duties. Title 17 U.S.C. 105 provides that copyright protection under this title is not available for any work of the United States Government. Title 17 U.S.C. 101 defines a U.S. Government work as work prepared by a military service member or employee of the U.S. Government as part of that person’s official duties.
